# Betalain production is possible in anthocyanin-producing plant species given the presence of DOPA-dioxygenase and L-DOPA

**DOI:** 10.1186/1471-2229-12-34

**Published:** 2012-03-12

**Authors:** Nilangani N Harris, John Javellana, Kevin M Davies, David H Lewis, Paula E Jameson, Simon C Deroles, Kate E Calcott, Kevin S Gould, Kathy E Schwinn

**Affiliations:** 1 New Zealand Institute for Plant & Food Research Limited, Private Bag 11-600, Palmerston North, New Zealand; 2 School of Biological Sciences, University of Canterbury, Private Bag 4-800, Christchurch, New Zealand; 3 Victoria University of Wellington, PO Box 600, Wellington 6140, New Zealand; 4 Commonwealth Scientific and Industrial Research Organization, Ecosystem Sciences, Urrbrea, South Australia 5064, Australia

## Abstract

**Background:**

Carotenoids and anthocyanins are the predominant non-chlorophyll pigments in plants. However, certain families within the order Caryophyllales produce another class of pigments, the betalains, instead of anthocyanins. The occurrence of betalains and anthocyanins is mutually exclusive. Betalains are divided into two classes, the betaxanthins and betacyanins, which produce yellow to orange or violet colours, respectively. In this article we show betalain production in species that normally produce anthocyanins, through a combination of genetic modification and substrate feeding.

**Results:**

The biolistic introduction of DNA constructs for transient overexpression of two different dihydroxyphenylalanine (DOPA) dioxygenases (DODs), and feeding of DOD substrate (L-DOPA), was sufficient to induce betalain production in cell cultures of *Solanum tuberosum *(potato) and petals of *Antirrhinum majus*. HPLC analysis showed both betaxanthins and betacyanins were produced. Multi-cell foci with yellow, orange and/or red colours occurred, with either a fungal DOD (from *Amanita muscaria*) or a plant DOD (from *Portulaca grandiflora*), and the yellow/orange foci showed green autofluorescence characteristic of betaxanthins. Stably transformed *Arabidopsis thaliana *(arabidopsis) lines containing *35S: AmDOD *produced yellow colouration in flowers and orange-red colouration in seedlings when fed L-DOPA. These tissues also showed green autofluorescence. HPLC analysis of the transgenic seedlings fed L-DOPA confirmed betaxanthin production.

**Conclusions:**

The fact that the introduction of DOD along with a supply of its substrate (L-DOPA) was sufficient to induce betacyanin production reveals the presence of a background enzyme, possibly a tyrosinase, that can convert L-DOPA to *cyclo*-DOPA (or dopaxanthin to betacyanin) in at least some anthocyanin-producing plants. The plants also demonstrate that betalains can accumulate in anthocyanin-producing species. Thus, introduction of a DOD and an enzyme capable of converting tyrosine to L-DOPA should be sufficient to confer both betaxanthin and betacyanin production to anthocyanin-producing species. The requirement for few novel biosynthetic steps may have assisted in the evolution of the betalain biosynthetic pathway in the Caryophyllales, and facilitated multiple origins of the pathway in this order and in fungi. The stably transformed *35S: AmDOD *arabidopsis plants provide material to study, for the first time, the physiological effects of having both betalains and anthocyanins in the same plant tissues.

## Background

The variety of colours observed in flowers, fruits and vegetative tissues in plants are due to the presence of chromogenic plant secondary metabolites [1,2]. These pigments serve diverse functions including photosynthesis and the protection of the photosynthetic machinery, attraction of pollinators and seed dispersers, and protection against biotic and abiotic stresses [1,3]. In addition to their biological functions, plant pigments are also of much interest regarding their possible beneficial effects on human health, their use as natural colorants and their aesthetic value in ornamental and food crops [4]. Non-chlorophyll plant pigments predominantly belong to two groups: flavonoids and carotenoids. Within the flavonoids, anthocyanins are the most significant type, providing a range of colours including orange, red, pink, mauve, purple and blue. However, in certain families within the order Caryophyllales, another class of pigments, the betalains, replaces the anthocyanins [2,5,6]. Betalains are only present in the order Caryophyllales and some fungi. They occur in most families of the Caryophyllales, but species of at least two families accumulate anthocyanin pigments instead [7]. The basis of this differentiation is unknown, but may represent an initial evolution of betalain biosynthesis in an ancestor of the core Caryophyllales and then its subsequent loss on different occasions [7].

No plant has yet been found that produces both betalain and anthocyanin pigments [5-8]. This mutually exclusive nature of the betalain and anthocyanin production in the plant kingdom is a curious phenomenon and the evolutionary and biochemical mechanisms for this restriction are unknown [5-7].

**Figure 1:**
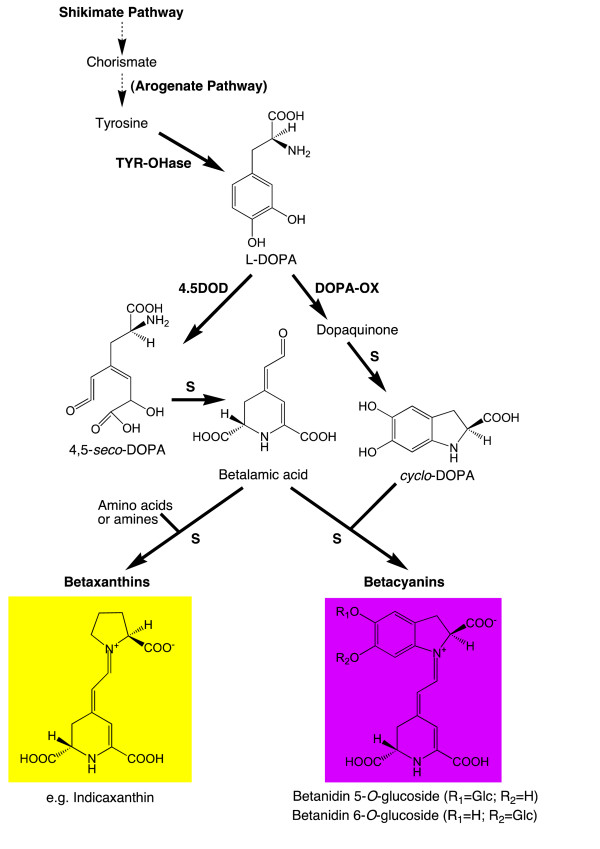
**Schematic of the proposed biosynthesis pathway of betalains**. Abbreviations are; 4,5DOD, DOPA-4,5-dioxygenase; DOPA-OX, DOPA oxidase; S, spontaneous conversion; TY-OHase, tyrosine hydroxylase. The steps for formation of the betacyanin glycosides are not shown.

There are two major types of betalains, the red-purple betacyanins and the yellow/orange betaxanthins, both of which accumulate in the vacuole. The betaxanthins also emit green autofluorescence, which is not seen with the betacyanins [9-11]. While the production of flavonoids and carotenoids has been extensively studied and metabolically engineered in a variety of species, betalain biosynthesis has yet to be fully characterised [1,2]. The betalain biosynthetic pathway is relatively simple with putatively only a few reactions that are enzyme catalysed (Figure 1). The initial biosynthetic step is the hydroxylation of tyrosine to L-3,4-dihydroxyphenylalanine (DOPA), attributed to the activity of a tyrosinase, although the exact role (if any) of tyrosinase in betalain synthesis has yet to be resolved [5,6,12,13] Cleavage of the cyclic ring of L-DOPA by DOPA-4,5-dioxygenase (DOD) forms an unstable *seco*-DOPA intermediate, which is thought to spontaneously convert to betalamic acid. The formation of betaxanthins occurs spontaneously from the condensation of betalamic acid with amines/amino acids [14], probably in the vacuole. The classic model of betacyanin biosynthesis involves the condensation of betalamic acid with (most commonly) *cyclo*-DOPA, which again is a likely spontaneous step. In this model, *cyclo*-DOPA is formed from L-DOPA through an oxidation reaction that also has been attributed to tyrosinase activity [5,6,13] The conversion proceeds via an unstable dopaquinone intermediate, which spontaneously cyclizes to form *cyclo*-DOPA. Betacyanins are generally *O*-glycosylated (at the C-5 or C-6) and frequently subsequently acylated. The aglycone product of betalamic acid and *cyclo*-DOPA condensation is termed betanidin and the 5-*O*-glucosylated form betanin, as with the anthocyanidin/anthocyanin convention. The timing of the glycosylation, regarding whether it occurs on *cyclo*-DOPA or betanidin, has been debated [15,16].

The only betalain biosynthetic enzymes characterised at the molecular level to date are for DOD and some of the glycosyltransferase activities. The first plant *DOD *gene characterised was from *Portulaca grandiflora*, and it defined a novel plant gene family of non-haem dioxygenases [2,11]. The plant dioxygenase is phylogenetically unrelated to DOD from the fungi *Amanita muscaria*, although the fungal sequence can complement betalain production in flowers of a *P. grandiflora dod *mutant [17]. The plant DOD carries out only the 4,5-cleavage of DOPA to yield betalamic acid, whereas the fungal enzyme can also conduct 2,3-extradiol cleavage of DOPA, yielding the yellow pigment muscaflavin [18]. Three cDNAs encoding proteins with relevant *O*-glycosylation activity have been identified, two that use both betanidin and flavonoids as substrates [19,20] and one that uses *cyclo*-DOPA [16].

Studies seeking to understand the genetic basis of the mutual exclusion of betalains and anthocyanins have focused on establishing the extent of the retention of the anthocyanin biosynthetic pathway in betalain producing species [21-23]. Flavonoids are present in betalain producing species, including flavonols and proanthocyanidins, and functional genes have been identified for the flavonoid biosynthetic enzymes chalcone synthase, dihydroflavonol 4-reductase and anthocyanidin synthase [21-23]. This suggests that the lack of anthocyanin production in betalain-producing species may be due to a lack of transcriptional activation of all the necessary biosynthetic genes [7,23], although a hypothesis based on repressive interaction between anthocyanin and betalain metabolites and the biosynthetic enzymes has also been suggested [24].

Our aim in this study was to determine whether betalain production is possible in anthocyanin-producing species. Using genetic transformation and feeding of pathway intermediates, we have examined what the minimum number is of biosynthetic steps that must be introduced into an anthocyanin-producing species to allow betalain production, and whether betalains can accumulate to significant levels in such species. Stable or transient transgene expression was used with DOD cDNAs from the fungus *A. muscaria *and the plant *P. grandiflora*, introduced into *Arabidopsis thaliana *(arabidopsis) plants, *Solanum tuberosum *(potato) cell cultures and *Antirrhinum majus *(antirrhinum) petals. These are representatives of the asterids and rosids, the two major clades of eudicots.

## Results and discussion

### Betalain biosynthesis in potato cell cultures by transient expression of *DOD*

**Figure 2:**
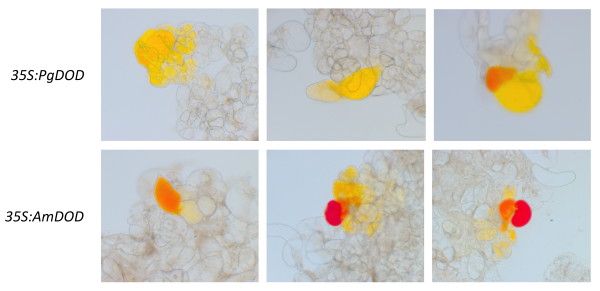
**Pigment production in potato cell suspension cultures biolistically transformed with *35S: PgDOD *or *35S: AmDOD *and fed 10 mM L-DOPA**. Examples are shown of cells with the resulting yellow to orange (*35S: PgDOD*) or yellow to red (*35S: AmDOD*) pigmentation.

Potato cell suspension cultures were transformed using particle bombardment with *35S: green fluorescent protein *(*GFP*) or constructs having either the *P. grandiflora DOD *cDNA (*35S: PgDOD*) or the *A. muscaria DOD *(*35S: AmDOD*) driven by the CaMV35S promoter, and examined for betalain production following feeding with L-DOPA. In cells transformed with *35S: GFP*, GFP was detected in the cells 24 h after biolistic transformation but no pigmentation was apparent (data not shown). In the cells transformed with either *35S: PgDOD *or *35S: AmDOD *and fed L-DOPA, pigmented multi-celled clusters were apparent within 24 h post-bombardment (Figure 2). Both *35S: AmDOD *and *35S: PgDOD *resulted in yellow and orange cell clusters. In addition, *35S: AmDOD *also produced cells with red pigmentation. The yellow and red pigmentation suggests that betaxanthin and betacyanin production, respectively, had been conferred to the cells. Indeed, observation of the yellow pigmented areas under blue light showed the autofluorescence characteristic of betaxanthin pigments (data not shown).

### Betalain biosynthesis in antirrhinum petals by transient expression of *DOD*

**Figure 3:**
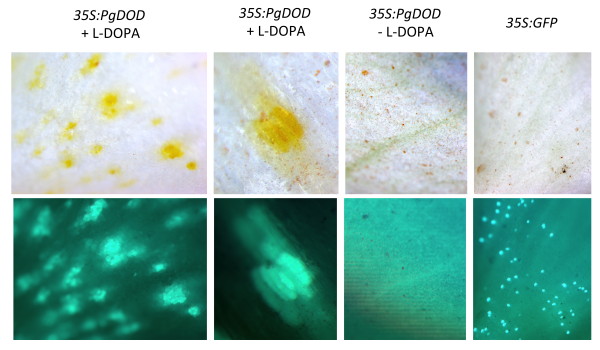
**Betaxanthin production in antirrhinum petals transiently transformed with *35S: PgDOD *or *35S: GFP***. Plasmid constructs containing *35S: PgDOD *or *35S: GFP *were introduced into the adaxial epidermis of antirrhinum petals using particle bombardment. At 24 h after bombardment some *35S: PgDOD *bombarded petals were infiltrated with 10 mM L-DOPA, while the other petals were infiltrated with water, and incubated for a further 24 h before observation. Representative petals are shown as viewed under white light (upper row) or blue light (lower row). The yellow, multi-cell foci for *35S: PgDOD *petals fed with L-DOPA are shown at two magnifications.

**Figure 4:**
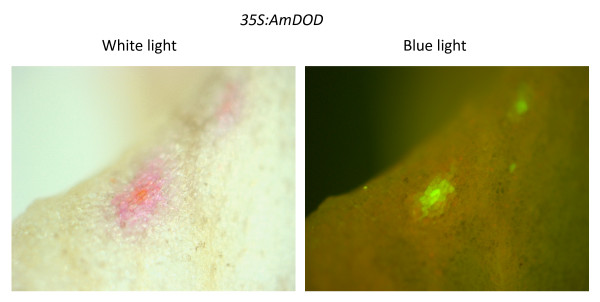
**Pigment production in antirrhinum petals transiently transformed with *35S: AmDOD***. A plasmid construct containing *35S: AmDOD *was introduced into the adaxial epidermis of antirrhinum petals using particle bombardment. At 24 h after bombardment the bombarded petals were infiltrated with 10 mM L-DOPA and incubated for a further 24 h before observation. A representative petal is shown as viewed under white light (left) or blue light (right).

The adaxial surface of dorsal petals of antirrhinum was transformed using particle bombardment with *35S: PgDOD *and examined for betalain production with or without infiltration of the petals with L-DOPA. Petals biolistically transformed with *35S: GFP *vector and fed with L-DOPA were used as an additional control. Antirrhinum lines having mutations in flavonoid production were used to provide anthocyanin-free petal backgrounds upon which to observe any pigment production. No pigments were visible in *35S: GFP *shot tissue or in petals transformed with *35S: PgDOD *but not infiltrated with L-DOPA, although positive GFP foci were apparent (Figure 3). The *35S: PgDOD *petals that were infiltrated with L-DOPA had numerous, multi-celled yellow foci 24 h after infiltration (48 h after bombardment) (Figure 3), indicating betaxanthin production. The yellow foci did indeed have the strong green autofluorescence typical of betaxanthins when observed under blue light (Figure 3). When using the *35S: AmDOD *construct, both yellow/orange and pink/red multi-celled foci occurred (Figure 4), although the pink/red foci were present only inconsistently (data not shown). Similarly, pink foci were sometimes present in some replicate experiments using *35S: PgDOD *(data not shown). The pink colouration indicates betacyanin production. The pink regions did not show strong autofluorescence while the central cells did, indicating possible betacyanin accumulation around a central region containing both betacyanins and betaxanthins.

**Figure 5:**
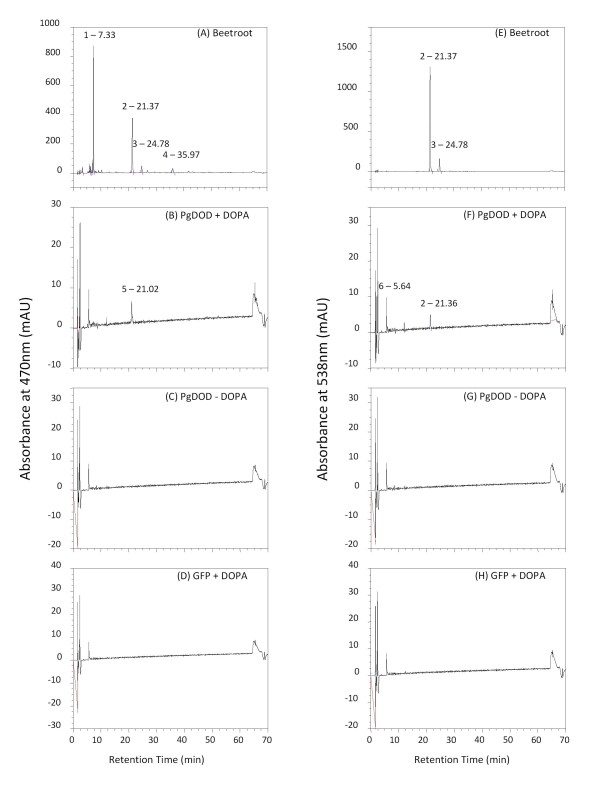
**HPLC analysis of pigments produced in antirrhinum petals transiently transformed with *35S: PgDOD *or *35S: GFP***. HPLC analysis was conducted on the *35S: PgDOD *and *35S: GFP *antirrhinum petal material shown in Figure 3. Absorbance was monitored at 470 nm for betaxanthins or 538 nm for betacyanins. Chromatograms are for extracts from beetroot root (A and E), *35S: PgDOD *antirrhinum infiltrated with L-DOPA (B and F), *35S: PgDOD *antirrhinum without L-DOPA infiltration (C and G), *35S: GFP *antirrhinum infiltrated with L-DOPA (D and H). The retention times of the major peaks are shown, and these were tentatively identified as per Table 1.

HPLC analysis (LC-DAD) was used to examine the nature of the yellow pigments produced following bombardment with *35S: PgDOD*. The ridge region of the petals was chosen for HPLC analysis, to ensure a similar region was sampled in each case. Betaxanthins and betacyanins have absorbance maxima at around 470 nm and 538 nm, respectively [25], and peak profiles were examined at these two wavelengths (Figure 5). No betalain-related peaks were detected with HPLC analysis of the *35S: GFP *petal tissue or with *35S: PgDOD *petals that were not infiltrated with L-DOPA. Petals transformed with *35S: PgDOD *and fed L-DOPA showed distinct peaks at both 470 nm and 538 nm. The compounds represented by the peaks were putatively identified by comparison of HPLC retention times and spectral data against those of a standard extract (from beetroot, Figure 5A and 5E) and reported spectral data [14,25,26]. The beetroot extract showed the expected peaks for the betaxanthin vulgaxanthin I (Peak 1) and the betacyanins betanin (betanidin 5-*O*-glucoside, Peak 2) and isobetanin (Peak 3) (Table 1). The *35S: PgDOD *L-DOPA fed samples showed a small amount of betanin (Peak 2; Figure 5F) and an unknown peak (Peak 5, 21.02 min; Figure 5B) that was possibly dopaxanthin (Table 1). Generally, the chromatograms at 538 nm showed the same patterns as at 470 nm except that vulgaxanthin I (Peak 1) and the putative dopaxanthin peak (Peak 5) were no longer detected, providing further evidence that Peak 5 is indeed a betaxanthin.

**Table 1:** Retention times and spectral maxima of the major betalain pigments detected in beetroot tissue (Peaks 1 to 3) or antirrhinum petal tissue bombarded with *35S: PgDOD *and infiltrated with L-DOPA (Peaks 4 and 5).

**Peak**^**a**^	Betalain	Retention Time (min)	λ_max _(nm)
1	Vulgaxanthin I	7.33	259, 469

2	Betanin	21.37	269, 534

3	Isobetanin	24.78	269, 534

4	Unknown betaxanthin	35.97	265, 464

5	Unknown betaxanthin^b^	21.02	241, 470

HPLC absorbance traces are shown in Figure 4

^a^The peak present at 5.64 min in antirrhinum tissue was present in all samples, both control and transgenic; its spectral data is not consistent with that of betalain pigments therefore it was excluded from the analysis.

^b^Possibly dopaxanthin

Antirrhinum can produce yellow pigments in the petal face and throat naturally. These pigments are aurones and a product of the flavonoid pathway. In addition to the spectral data (aurones have spectra maxima in the range 390-430 nm), there is other evidence that the yellow pigments observed following *35S: PgDOD *bombardment and L-DOPA feeding are unlikely to be aurones; aurones are not present normally in the dorsal petals of the antirrhinum line used (JI19), and no yellow pigments were observed without infiltration with L-DOPA.

Despite a lack of the red colouration seen following bomardment, the *35S: PgDOD *antirrhinum samples analysed by HPLC did indeed contain small amounts of betacyanin (betanin; Figure 5F). Also, the presence of betacyanin is inferred in the *35S: AmDOD *expressing potato cells, given the red colouration of some foci. How betacyanin production can occur in non-betalain species through the introduced DOD acting on the supplied L-DOPA is not clear. The product of DOD action on L-DOPA is betalamic acid (Figure 1). Betaxanthins can be produced by spontaneous reactions of the DOD reaction end-product, betalamic acid, with amino acids or amines. However, betacyanin formation requires *cyclo*-DOPA, the formation of which from L-DOPA by oxidation has been attributed to the activity of a tyrosinase [6]. As tyrosinases are frequently present in plant cells it is possible that an endogenous enzyme with activity on L-DOPA occurs in most non-betalain producing species. Alternatively, betacyanin could be formed from endogenous tyrosinase activity on dopaxanthin [12]. The betacyanin detected in antirrhinum petals was likely betanin, an *O*-glycosylated betacyanin. This indicates that endogenous glycosyltransferases can act on the novel betacyanin substrates. Two *O*-glucosyltransferases have been characterized with activity on betanidin [19,20]. Both show sequence similarity to the *O*-glycosyltransferases involved in anthocyanin biosynthesis, and indeed, the betanidin 5-*O*-glucosyltransferase has activity with both betacyanins and flavonoids [20]. Thus, it may be the case that the endogenous flavonoid *O*-glucosyltransferases of antirrhinum are also able to act on betanidin (and/or *cyclo*-DOPA).

Biolistic transformation of both potato and antirrhinum with either *35S: PgDOD *or *35S: AmDOD *resulted in muli-celled foci producing betalains. It is unlikely that this would be due to movement of the DOD enzyme between cells as, similar to GFP (Figure 3), it is too large for passive intercellular movement. As the final betalain pigments accumulate within the vacuole, this suggests that some of the precursors migrate between cells. This is similar to the results of Mueller *et al. *[17] when they biolistically introduced *AmDOD *into different *P. grandiflora *mutant backgrounds. Single coloured cells seen within 18 h after bombardment developed into multi-cell foci by 48 h after bombardment [17]. It was suggested that betalains could have diffused through plasmodesmata to neighbouring cells. However, in contrast to these results, when complementation of the *P. grandiflora dod *mutant was conducted with PgDOD, only single cell foci occurred [11]. Furthermore, cell-specific betalain production is commonly observed in plants, such as in the epidermal cells of petals. In the case of our results, it is possible that high L-DOPA levels produced from tissue feeding allowed the movement of betalamic acid not just to the vacuole but also to neighbouring cells.

### Betalain pigment production by stable transformation of Arabidopsis

**Figure 6:**
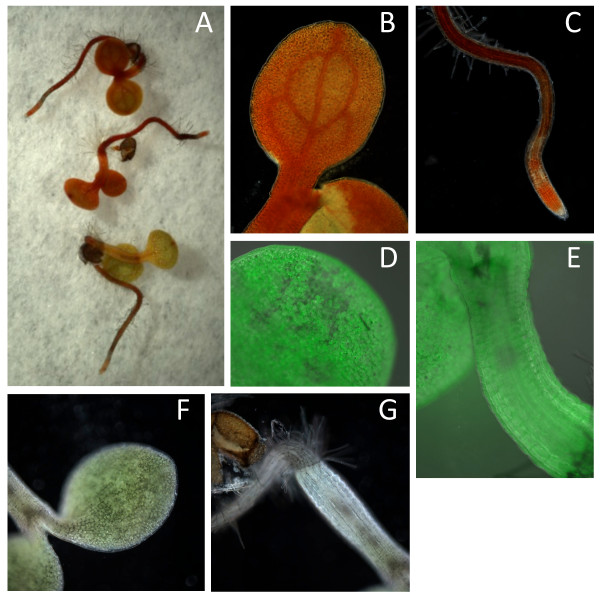
**Betaxanthin production in arabidopsis seedlings stably transformed with *35S: AmDOD***. *35S: AmDOD *seedlings were grown on moistened filter disks either with (**A **to **E**) or without (**F **and **G**) 10 mM L-DOPA. Those fed L-DOPA accumulated yellow to orange/red pigments (**A **to **C**) and under blue light showed the autofluorescence typical of betaxanthins, as illustrated for the cotyledon (**D**) and hypocotyl (**E**). The seedlings not fed L-DOPA did not produce coloured pigments in either their cotyledons (**F**) or hypocotyls (**G**).

**Figure 7:**
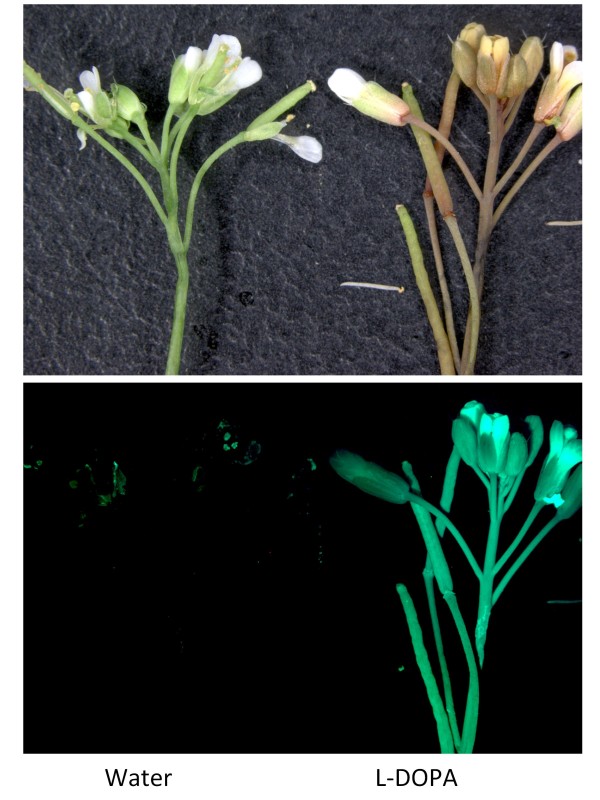
**Pigment production in inflorescences of arabidopsis plants stably transformed with *35S: AmDOD***. Inflorescences from *35S: AmDOD *arabidopsis line 6 were infiltrated with either water or 10 mM L-DOPA for 24 h and examined under white light (upper panel) or blue light (lower panel). Those fed L-DOPA accumulated yellow to orange pigments and under blue light showed the autofluorescence typical of betaxanthins. The inflorescences not fed L-DOPA did not produce visible coloured pigments and did not show autofluorescence.

Stably transformed arabidopsis plants were produced through *Agrobacterium*-mediated transformation with *35S: AmDOD*. T2 generation seedlings were checked for expression of the *35S: AmDOD *transgene (Additional file 1) and selected lines examined for their potential to produce betalains when fed with L-DOPA. When whole seedlings were fed L-DOPA, novel pigment production was visible within 12 h after feeding (Figure 6A-C), including in the etiolated root tissues and the hypocotyls. The colour ranged from pale yellow through to orange and dark orange-red. Under blue light, the pigments showed the green autofluorescence characteristic of betaxanthins (Figure 6D, E). When detached inflorescences from mature plants were fed L-DOPA pale yellow pigmentation was seen in all tissues 24 h after feeding, including the stem, petals and siliques, and this was accompanied by strong autofluorescence (Figure 7). *35S: AmDOD *seedlings or inflorescences not fed L-DOPA did not produce visible pigments after treatment and did not show significant autofluorescence under blue light (Figure 6F, G and Figure 7).

**Figure 8:**
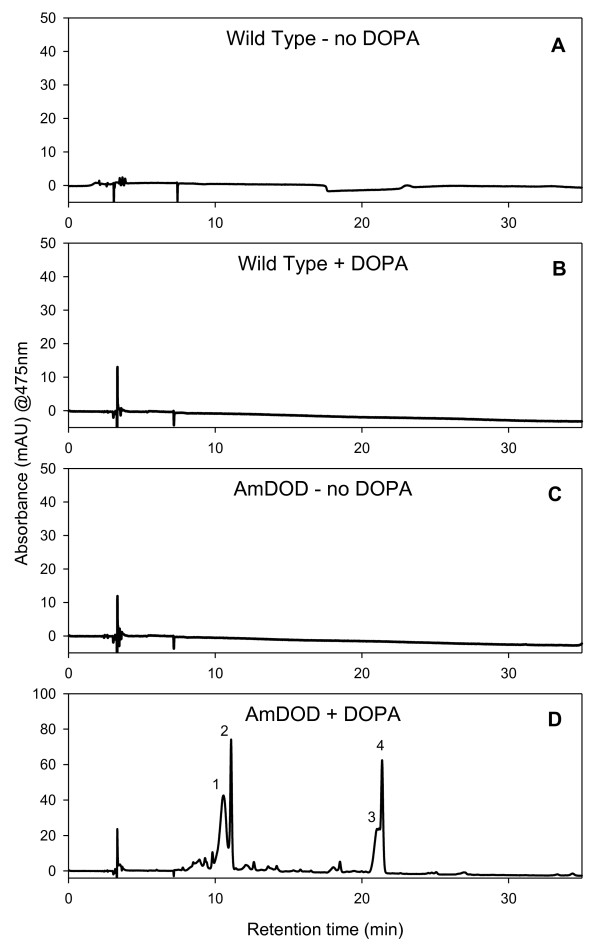
**HPLC analysis of pigments produced in arabidopsis seedlings stably transformed with *35S: AmDOD***. Seedlings, representatives of which are shown in Figure 6, were grown on moistened filter paper with or without the addition of 10 mM L-DOPA and the pigments present examined using HPLC. Samples from wild-type (non-transgenic) arabidopsis and from beetroot root were included for comparison. Absorbance was monitored at 475 nm for betaxanthins. The retention times of the major peaks are shown, and these were identified as betaxanthins per Table 2.

HPLC analysis was used to examine the nature of the pigments produced following L-DOPA feeding of the *35S: AmDOD *arabidopsis. Seedlings were fed L-DOPA and the entire seedling sampled for chemical analysis. HPLC analysis revealed several peaks present in the L-DOPA fed *35S: AmDOD *tissue but not in the control tissue (Figure 8). Four of these peaks were present in sufficient quantity to confirm that their spectral data and retention times were those characteristic of betaxanthins (Table 2).

**Table 2:** Retention times and spectral maxima of the major peaks detected by HPLC using samples from *35S: AmDOD *plants fed 10 mM L-DOPA

Peak	**Betalain**^**a**^	Retention Time (min)	λ_max _(nm)
1	Vulgaxanthin I^b^	10.4	268, 468

2	Unknown betaxanthin	11.6	262, 470

3	Unknown betaxanthin	20.4	264, 472

4	Unknown betaxanthin	21.7	262, 468

^a^Spectral data for all four peaks was generally consistent with data reported previously for betaxanthins, but no specific betaxanthin assignments were made.

^b^Peak one has a retention time and spectrum consistent with that of Vulgaxanthin I, as reported for extracts from beetroot [14] and as compared against a beetroot extract run in the same experimental set.

The presence of betaxanthins matched the strong autofluorescence observed in the seedlings under blue light. The failure to detect the presence of betacyanin was a surprise given the orange/red colouration of the seedlings and the ability of *DOD *to confer both betaxanthin and betacyanin production when transiently expressed in antirrhinum petals. In accordance with the HPLC results, while the seedlings did have an orange-red colour that would suggest betacyanin production, the autofluorescence indicated strong betaxanthin presence. Furthermore, the fed inflorescences off the mature plants did not have a red colouration. Thus it appears that a high level of betaxanthin production in the particular background colouration of the seedlings resulted in the orange-red colour. It is unlikely that anthocyanins are contributing to the orange-red colouration of seedlings, as L-DOPA treatment of non-transgenic seedlings did not induce visible pigment production.

## Conclusions

We report here methods to enable production of high-levels of betalains in the non-betalain, anthocyanin-producing, species antirrhinum, arabidopsis and potato, which represent both of the major clades of the eudicots. Betalains are not normally observed in anthocyanin-producing species, for unknown reasons.

The introduction of a single enzyme (DOD) and feeding of that enzyme's substrate (L-DOPA) were sufficient to induce both betaxanthin and betacyanin production. Although many steps in betalain production are suggested to be spontaneous, it is somewhat surprising that betacyanin production occurred. It indicates the background presence of an enzyme able to convert L-DOPA to *cyclo*-DOPA, or dopaxanthin to betacyanin/betanidin, and *O*-glycosyltransferases with activity against betanidin. Thus, it is likely that introduction of just two enzymes, DOD and the (currently uncharacterised) enzyme for conversion of tyrosine to L-DOPA, should be sufficient to confer betaxanthin and betacyanin production to anthocyanin-producing species. The requirement for few novel biosynthetic steps may have assisted in the initial evolution of this pathway in the Caryophyllales. A relatively simple mechanism for betalain biosynthesis would also support the multiple origins of the pathway in the Caryophyllales and in some fungi [7].

Given the extensive knowledge available on anthocyanin production and function in arabidopsis, the stably transformed *35S: AmDOD *arabidopsis plants should provide excellent material to study, for the first time, the physiological effects of having both betalains and anthocyanins in the same plant tissues. The large collection of mutant lines in arabidopsis should also allow aspects of betalain biosynthesis, such as vacuolar transport, to be examined.

## Methods

### Plant and cell culture material

*Antirrhinum majus *plants were grown under standard glasshouse conditions in Palmerston North, New Zealand. The glasshouse was heated at 15°C and vented at 25°C, without supplementary lighting. Two antirrhinum lines were used. Transformation with *35S: PgDOD *used line JI19, which produces aurones and flavones but not anthocyanins in the petals, as it is lacking in flavanone 3-hydroxylase activity (homozygous *incolorataII*). 35S: AmDOD transformation used either JI19 or *rosea^dorsea^*, which carries a mutation in the R2R3MYB gene *Rosea1 *and lacks anthocyanin production in the adaxial epidermis of the petals [27]. *Arabidopsis thaliana *transformation used the Colombia ecotype. Potato cell cultures were obtained by placing 2 cm^2 ^pieces of potato callus in 50 mL of modified liquid MS media [28] per cotton-plugged 250 mL flask. These cultures were incubated at room temperature (23-27°C) with shaking on an orbital shaker at 100 rpm in the dark. Subculturing was carried out every two weeks with 3 mL of the cell culture used to inoculate a fresh 50 mL of liquid media.

### PCR cloning of *P. Grandiflora *DOD cDNA and vector construction

A cDNA for the ORF of *DOD *was RT-PCR amplified from mRNA prepared from *P. grandiflora *betalain pigmented petals using primers designed to the published sequence [11]. The forward primer was 5'-AGTCAGAATCCATGGGTGTTGGGAAGGAA-3', with the first ATG matching the initiation ATG for DOD, and the reverse primer was 5'-AGTCATCTAGAATCATATGGAAGTGAACT-3', which incorporated an XbaI site. Standard PCR conditions were used with Taq Polymerase (New England Biolabs, Massachusetts, USA) and cycling parameters of 94°C for 4 min, then 30 cycles of 94°C for 30 s, 60°C for 30 s and 72°C for 1 min, with a final extension phase of 72°C for 7 min. The products were cloned into pGEM-T-Easy (Promega, Wisconsin, USA) and confirmed as *PgDOD *by DNA sequencing. The cDNA was then excised as an EcoRI/XbaI fragment and ligated into EcoRI/XbaI digested *pART7 *[29] to from the vector pPN314. The *35S: AmDOD *construct for particle bombardment, containing the *A. muscaria *4,5-DOPA dioxygenase cDNA driven by the CaMV35S promoter, was *pNcoDod *[17] (courtesy of Dr. Willibald Schliemann, Leibniz-Institute of Plant Biochemistry, Germany). The binary vector *pPN166 *for transformation into arabidopsis was constructed by taking the CaMV35S-cDNA region from *pNcoDod *as a PvuII fragment and cloning it into the NotI site (after flushing by end-filling) in the binary vector *pMLBART *(a gift from Dr Bart Janssen, Plant & Food Research). The *35S: GFP *construct was pPN93. Vectors were verified by restriction fragment analysis and/or DNA sequencing.

### Biolistic transformation

Antirrhinum particle bombardment experiments were performed as described in Shang *et al. *[30], with the following variations; the pressure setting was 300 or 400 kPa, the shooting distance 11-12 cm, and petals were bombarded twice. The final DNA concentration for DOD constructs was 1 or 2 μg DNA per mg of 1.0 μm gold particles. Controls included gold particles alone (no DNA) and *35S: GFP *(added at 0.4 μg DNA per mg of 1.0 μM gold particles). Prior to bombardment the petals were surface sterilised by immersion for 15 min in 10% (v/v) bleach containing a few drops of Tween-20, followed by three rinses in sterile water. The adaxial surface of the dorsal petals was bombarded. After bombardment, the plant materials were then cultured on 1/2 MS medium under 20 - 50 μmol m^-2 ^s^-1 ^light from Osram 36 W grolux fluorescent tubes (16 h photoperiod) at 25°C. At least two flowers were used for each construct per experiment, and each experiment was repeated at least twice.

Potato cell suspension cultures for particle bombardment-mediated transformation were prepared by filtering 3 mL of culture onto sterile filter paper and sub-culturing them on a solid media in tubs for 48 h prior to transformation. The biolistic parameters were the same as those used for the antirrhinum petals with exception that they were only bombarded once. A sterilised metal grid was placed over the cells, on top of the culture tub, to prevent displacement of the cells from the helium in-flow.

### Arabidopsis transformation

The floral spray method developed by Clough and Bent [31] was used for the transformation of arabidopsis. Seeds were harvested from the *Agrobacterium*-inoculated plants and transgenics selected by spraying germinating seedlings with glufosinate herbicide (Basta, Bayer Crop Sciences). Positive transformants were grown through to production of T2 seeds following self-pollination.

### L-DOPA feeding

Infiltration of antirrhinum petals with L-DOPA was carried out 24 h after biolistic transformation. The petals were placed in 10 mM L-DOPA solution and a brief vacuum (30-60 s) applied, until the solution boiled vigorously and the petals became translucent. Following infiltration excess solution was blotted off and the petals incubated on 1/2 MS medium under the same conditions as post-bombardment. Control petals were handled in the same manner but with sterile water substituted for L-DOPA. Petals were observed for betalain production after a further 24 h. Transformed potato cell cultures were fed with 10 mM L-DOPA solution by dispensing 1 mL of the solution on to the filter disks supporting the cells. The L-DOPA solution was applied to half of the samples immediately after transformation.

Germinated arabidopsis seedlings were fed with 10 mM L-DOPA by transfer of the filter paper containing the seedlings on to a plate containing L-DOPA solution. Seedlings were observed for betalain pigment production after a further 12 h. For feeding of inflorescences, six inflorescences were collected from *35S: AmDOD *line 6 and cut to 1 cm long. Three inflorescences were immersed in 10 mM L-DOPA solution and the other three immersed in water, and both sets were left for 2 min. The inflorescences were then placed into 1.5 ml Eppendorf tubes with either 0.5 ml of 10 mM L-DOPA solution or water, and the tubes left open on the bench for 24 h.

### PCR analysis

RT- PCR analysis for *DOD *transgene expression in *35S: AmDOD *arabidopsis plants used total RNA extracted from leaf tissue following the RNeasy protocol of the Qiagen RNeasy mini kit. Five rosette leaves were sampled from each of six individual T2 lines, as well as a non-transgenic wild type line. Following DNAse I treatment 500 ng of RNA from each sample was used to generate cDNA using the Roche Transcriptor First Strand cDNA Synthesis Kit. PCR used Taq polymerase (Roche, New Zealand) and the following primers: DODF1 5'-CATACTACCATGTCCACCAAG-3', DODF2 5'-AGCACTGCTTCTATATCGTC-3', Act2S 5'-TCCCTCAGCACATTCCAGCAGAT-3', Act2AS 5'-AACGATTCCTGGACCTGCCTCATC-3'. The Actin primers correspond to the arabidopsis *Actin2 *gene (AT3G18780). The thermocycling conditions were 94°C for 2 min and 25 cycles of 94°C for 20 s, 55°C for 30 s and 72°C for 30 s. The PCR products were separated on a 1% (w/v) TBE agarose gel containing ethidium bromide and visualised using UV-illumination. PCR products were also cloned and sequenced to confirm that the target PCR product was being amplified.

### HPLC analysis

For arabidopsis analysis whole seedlings were extracted. For antirrhinum analysis, to ensure that similar petal regions were being compared, the ridge region of the biolistically transformed petals was excised away from the remainder of the petals for chemical analysis. The ridge is a distinct raised area that divides the lobes and throat of the dorsal petals. Ridge tissue from between three and six petals was pooled to give total sample fresh weights of 60 to 300 mg. Absorbance at 470 nm and 538 nm was used for detection of betaxanthins or betacyanins, respectively. Beetroot (20 mg freeze dried sample of red root) was used as a standard source of betalains, and strong peaks with the expected retention times and spectra data were observed.

Each sample was extracted three times in 1 ml of 80% (v/v) methanol containing 50 mM sodium ascorbate, as described in Schliemann *et al. *[14], with one overnight extraction at 4°C. The samples were centrifuged for 4 min at 10,000 rpm, the supernatant removed and the pellet re-extracted in the next 1 ml of 80% methanol with ascorbate. The supernatants once removed, were combined to give the crude extract. The extract was dried in vacuo on a Savant SC210 Speedvac to near dryness, then freeze dried overnight to complete dryness. Extracts were resuspended in MilliQ water and made up to a final volume of 500 μl. The extracts were syringe filtered through a 0.45 um nylon filter as per Svenson *et al. *[26] and the pigments analysed by high performance liquid chromatography (HPLC). The analysis of the antirrhinum samples used a Dionex 3000 Ultimate solvent delivery system with a Phenomenex Luna (5 μm, 150 × 4.6 mm) RP-18 endcapped column (column temperature 30°C) and a Dionex 3000 Diode Array Detector (DAD). Elution (1 ml min^-1^) was performed using a solvent system comprising solvent A [1.0% formic acid in water] and solvent B [80% acetonitrile in water] and a linear gradient starting with 100% A, decreasing to 80% A at 62 mins, and then a linear gradient to 100% B at 67 mins, remaining at 100% B for a further 3 min, then returning to initial conditions. Betaxanthins were detected at 470 nm and betacyanins at 538 nm [25]. The analysis of the arabidopsis samples used the solvent system of Schliemann *et al. *[14]. It was conducted on a Hewlett Packard HP 1100 with two Merck Chromolith analytical columns and a C-18 guard column.

## Authors' contributions

NNH conducted the AmDOD biolistic experiments in antirrhinum, produced and contributed to the analysis of the arabidopsis plants, and contributed to writing of the manuscript; JJ made pPN314 and conducted the biolistic experiments in potato; KMD conducted the PgDOD biolistic experiments in antirrhinum and wrote the main manuscript draft; DHL conducted the HPLC analysis of biolistically transformed material and contributed to the manuscript draft; PEJ contributed to supervision of JJ and NNH and project design; SCD assisted with cell culture experiments and supervision of JJ; KEC conducted PCR analysis and inflorescence feeding for the arabidopsis plants; KSG contributed to supervision of KEC. KES conceived and coordinated the study, supervised NNH and JJ, and contributed to analysis of the results and writing of the manuscript. All authors read and approved the final manuscript.

## Supplementary Material

Additional file 1 **PCR analysis for *DOD *transgene expression in *35S: AmDOD *arabidopsis plants**. Total RNA was extracted from six lines of *35S: AmDOD *arabidopsis, as well as a non-transgenic wild type line, and analysed for *DOD *transcript levels using RT-PCR. PCR primers for an endogenous actin gene were used as a positive control for RNA/cDNA integrity. PCR products were separated on a 1% (w/v) agarose gel containing ethidium bromide and visualised using UV-illumination. (PPT 356 kb). Click here for file
