# Supplementary figures and images for: Betalain production is possible in anthocyanin-producing plant species given the presence of DOPA-dioxygenase and L-DOPA
Source: BMC Plant Biol. 2012 Mar 12;12:34. doi: 10.1186/1471-2229-12-34 (PMC3317834; doi:10.1186/1471-2229-12-34)

## Slide 1
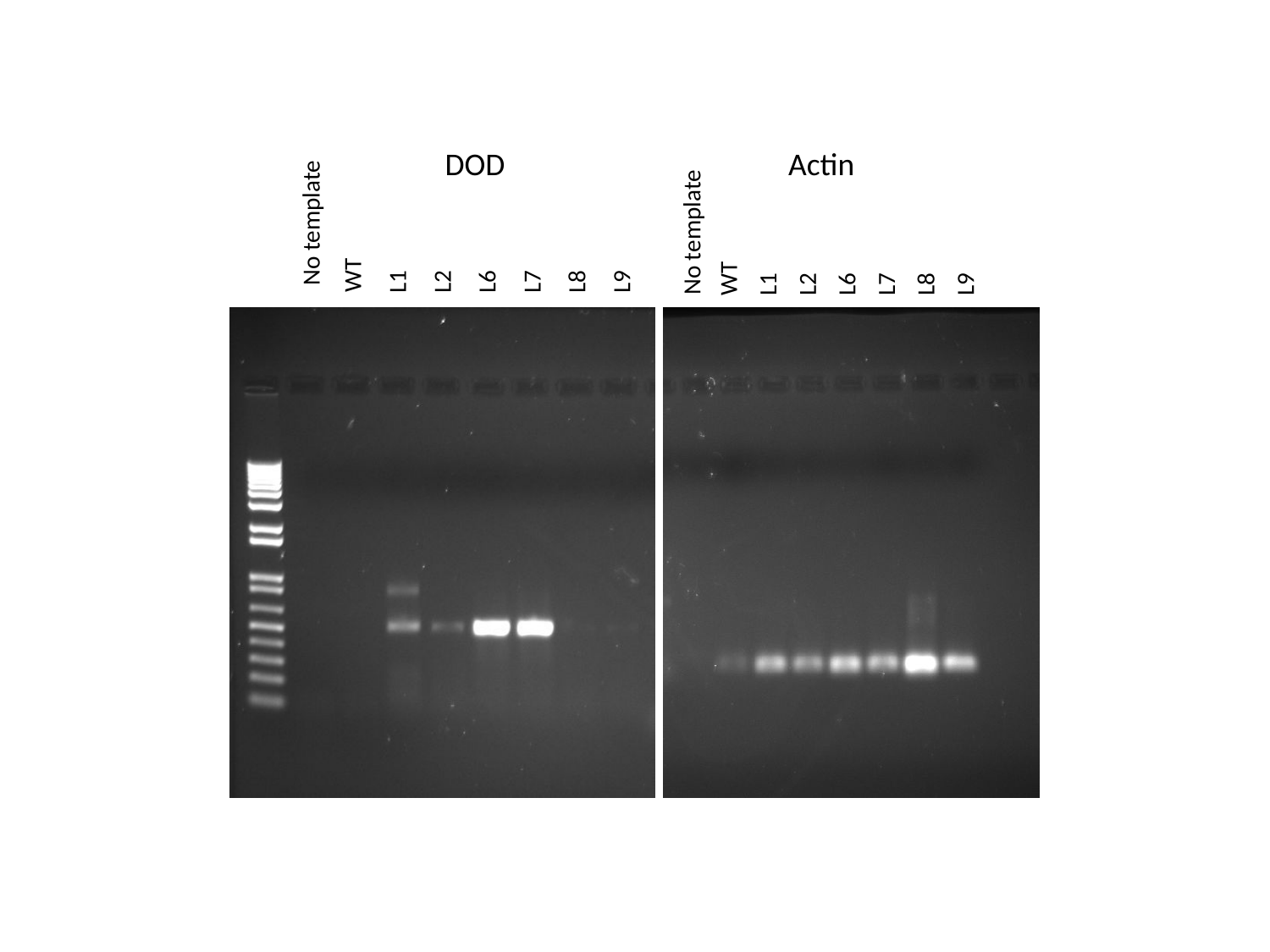

DOD
Actin
No template
WT
L2
L6
L7
L8
L9
L1
No template
WT
L2
L6
L7
L8
L9
L1

Supplement: Additional file 1 — PCR analysis for DOD transgene expression in 35S: AmDOD arabidopsis plants. Total RNA was extracted from six lines of 35S: AmDOD arabidopsis, as well as a non-transgenic wild type line, and analysed for DOD transcript levels using RT-PCR. PCR primers for an endogenous actin gene were used as a positive control for RNA/cDNA integrity. PCR products were separated on a 1% (w/v) agarose gel containing ethidium bromide and visualised using UV-illumination. (PPT 356 kb). [file 1471-2229-12-34-S1.PPT]
